# Management of testicular tumours in patients with undescended testes— a challenging but rewarding task: experience from a tertiary care cancer centre in India

**DOI:** 10.3332/ecancer.2023.1521

**Published:** 2023-03-20

**Authors:** Arnav Tongaonkar, Vijai Simha, Nandini Menon, Vanita Noronha, Ganesh Bakshi, Vedang Murthy, Santosh Menon, Nilesh Sable, Rahul Krishnatry, Palak Popat, Mahendra Pal, Gagan Prakash, Archi Agarwal, Bhagyashri Shivaji Jadhav, Kumar Prabhash, Amit Joshi

**Affiliations:** 1 Department of Medical Oncology, Tata Memorial Centre, Homi Bhabha National University (HBNI), Mumbai 400012, India; 2 Consultant Medical Oncologist, Sri Shankara Cancer Hospital and Research Centre, Bangalore 560004, India; 3 Consultant Surgical Oncologist, Hinduja Hospital, Mumbai 400016, India; 4 Department of Radiation Oncology, Tata Memorial Centre, Homi Bhabha National University (HBNI), Mumbai 400012, India; 5 Department of Pathology, Tata Memorial Centre, Homi Bhabha National University (HBNI), Mumbai 400012, India; 6 Department of Radiodiagnosis and Imaging, Tata Memorial Centre, Homi Bhabha National University (HBNI), Mumbai 400012, India; 7 Department of Surgical Urology, Tata Memorial Centre, Homi Bhabha National University (HBNI), Mumbai 400012, India; 8 Department of Nuclear Medicine, Tata Memorial Centre, Homi Bhabha National University (HBNI), Mumbai 400012, India

**Keywords:** testicular tumour, undescended testes, germ cell tumour, management, cryptorchidism

## Abstract

**Objective:**

Primary objective: To study patients’ clinical profile and outcomes with germ cell tumours developing in undescended testes.

**Materials and methods:**

Case records of patients enlisted in the prospectively maintained ‘testicular cancer database’ at our tertiary cancer care hospital from 2014 to 2019 were retrospectively reviewed. Any patient who presented with testicular germ cell tumour with a documented history/diagnosis of undescended testes, whether surgically corrected or not, was considered for this study. The patients were managed along the standard lines of treatment for testicular cancer. We evaluated clinical features, difficulties and delays in diagnosis and complexities in management. We evaluated event-free survival (EFS) and overall survival (OS) using the Kaplan–Meier Method.

**Results:**

Fifty-four patients were identified from our database. The mean age was 32.4 years (median age 32, range: 15–56 years). Seventeen (31.4%) had developed cancer in orchidopexy testes, and 37 (68.6%) presented with testicular cancer in uncorrected cryptorchid testes. The median age at orchidopexy was 13.5 years (range: 2–32 years). The median time from symptom onset to diagnosis was 2 months (1–36 months). There was a delay in the initiation of treatment of more than 1 month in 13 patients, with the longest delay being 4 months. Two patients were initially misdiagnosed as gastrointestinal tumours. Thirty-two (59.25%) patients had seminoma, and 22 (40.7%) patients had non-seminomatous germ cell tumours (NSGCT). Nineteen patients had metastatic disease at presentation. Thirty (55.5%) patients underwent orchidectomy upfront while in 22 (40.7%) patients, orchidectomy was done after chemotherapy. The surgical approach included high inguinal orchidectomy, exploratory laparotomy or laparoscopic surgery per the clinical situation. Post-operative chemotherapy was offered as clinically indicated. At a median follow-up of 66 months (95% CI: 51–76), there were four relapses (all NSGCT) and one death. The 5-year EFS was 90.7% (95% CI: 82.9–98.7). The 5-year OS was 96.3% (95% CI: 91.2–100)

**Conclusions:**

The tumours in undescended testes, particularly those without prior orchiopexy, often presented late and with bulky masses, requiring complex multidisciplinary management. Despite the complexity and challenges, our patient’s OS and EFS matched that of patients with tumours in normally descended testes. Orchiopexy may help in earlier detection. In the first such series from India, we show that testicular tumours in the cryptorchid are also as curable as the germ cell tumours developing in the descended testis.

A multidisciplinary disease management group with expertise in managing complex cases is crucial for a favourable outcome in these groups of patients. We also found that orchiopexy done even later in life confers an advantage in terms of early detection in a subsequently developing testicular tumour.

## Introduction

The male gonads form in the intermediate mesenchyme of the urogenital ridge in the abdomen and begin the descent at 12 weeks of gestation, reaching the inguinal canal by 33 weeks. Cryptorchidism results when one or both testes fail to reach the scrotum at birth and occurs in 2%–4% of the population. Cryptorchidism confers a higher risk of testicular tumours with a pooled relative risk of 2.9 in a meta-analysis [1]. Walsh *et al* [2] in their meta-analysis showed that the risk of testicular cancer in those with undescended testes was up to 6 times greater in those whom pre-pubertal orchidopexy was not performed [2]. The ideal time is before 2 years of age. However, due to various socio-economic reasons, the rates of ‘*timely orchiopexy*’ are significantly less, with the median age at orchiopexy being around 4 years [3, 4] across different parts of the world [4, 5]. In developing countries, >50% of children are detected to have cryptorchidism beyond 8 years, decreasing the effectiveness of cryptorchidism in preventing testicular cancer [6].

The challenges in managing testicular cancer in cryptorchid testis, presenting directly with cancer, have been sparsely described in the literature [7–9], especially those in an intra-abdominal or pelvic location.

In this study, we included only testicular cancers which occur in undescended testis, whether surgically corrected or uncorrected. We studied the clinical presentation, diagnosis and treatment complexities in both sets of patients. The role of upfront chemotherapy in managing these patients was also highlighted.

## Materials and methods

**Figure 1.**
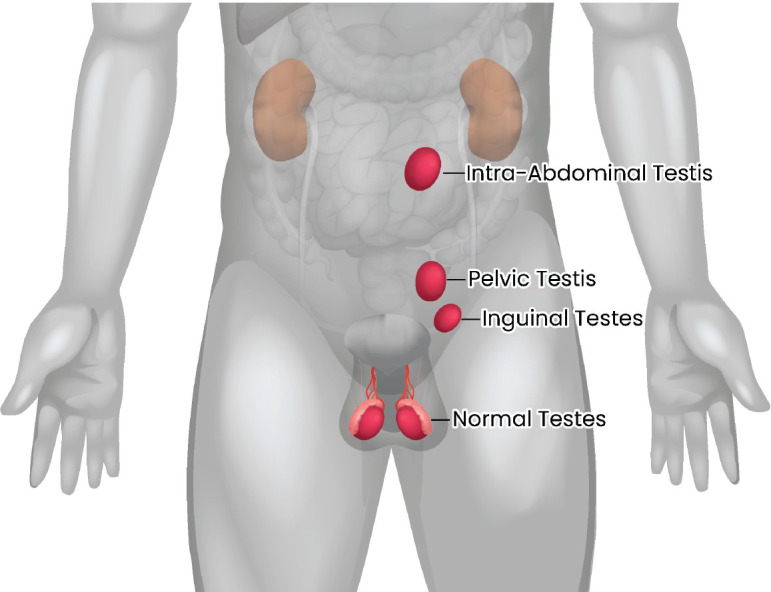
Location of undescended testes.

Our hospital is a high-volume premier tertiary care cancer institute in India. We maintain a Testicular Cancer database that is updated prospectively. Case records of patients enlisted from 2014 to 2019 in the database were reviewed. Germ cell tumours occurring in patients with a history of undescended testes, with or without prior orchiopexy, were considered for this study. The location of the testis was considered intra-abdominal if the testicular mass’s epicentre was above the pelvic brim level and considered pelvic when the epicentre was below the pelvic brim but outside the inguinal canal (Figure 1). All newly diagnosed testicular cancer patients were discussed in a multidisciplinary tumour board before beginning treatment. The serum tumour markers: β-human chorionic gonadotropin, α-fetoProtein and lactate dehydrogenase were obtained in all the patients. The staging was done as per the American Joint Committee on Cancer (AJCC), including the ‘S’ staging system. Risk stratification was done as per the International Germ Consensus (IGCC) risk classification. In patients unsuitable for upfront orchidectomy, the tumour markers at baseline were considered for treatment decisions.

The date of diagnosis was defined as the date of first clinical diagnosis, based on the detection of raised tumour markers or the first histologically proven presence of a testicular tumour, whichever was earlier. Patients underwent high inguinal orchidectomy or abdominal orchidectomy (if feasible) as a therapeutic procedure. Patients with pelvic or intra-abdominal testis underwent orchidectomy by laparotomy or laparoscopy as clinically suitable. Patients who could not undergo surgery upfront were treated with upfront chemotherapy before orchidectomy. The treatment post-orchidectomy was according to the stage and IGCC risk of the tumours. Bleomycin, Etoposide, Cisplatin (BEP) regimen was the standard chemotherapy regimen used. Other regimens like Etoposide, Ifosfamide, Cisplatin (VIP) and Etoposide-Cisplatin were used when bleomycin was to be avoided: e.g., in heavy smokers or patients with multiple pulmonary metastases. Patients with stage I seminoma was given 1–2 cycles of single-agent carboplatin as appropriate. Radiation was also considered in Stage I and IIA seminoma. At the end of chemotherapy, patients underwent reassessment by imaging and tumour markers for the presence of residual disease. PET-CT imaged patients with seminoma after 10–12 weeks of chemotherapy, and non-seminomatous germ cell tumours (NSGCT) were imaged 4–6 weeks after the last chemotherapy. The patients were reviewed post-chemotherapy in a multi-disciplinary clinic for a treatment plan. Patients with the residual disease were considered for retroperitoneal lymph node dissection (RPLND) ± orchidectomy or metastasectomy as clinically indicated. The combination of orchidectomy, chemotherapy and RPLND or metastasectomy for the residual disease was defined as ‘*first-line’* treatment.

The patients were followed up with 3 monthly assessments of tumour markers and 6 monthly imaging after treatment as per institutional guidelines. Patients with unresectable disease, persistently raised tumour markers or imaging findings (CT/PET CT) were considered to have persistent disease. These cases were discussed in our multi-disciplinary joint clinics for planning further therapy. Patients with significant residual disease or progression post the primary therapy or those who recurred after primary treatment were given second-line salvage chemotherapy with VIP or paclitaxel, ifosfamide, cisplatin (TIP) chemotherapy.

The clinical data were presented as descriptive statistics of the overall population. ‘Event’ was defined as patients with persistent disease after first-line treatment or those who had a recurrence after achieving complete remission from first-line treatment. Event-free survival (EFS) was defined as the time from diagnosis to the first ‘event’. Overall survival (OS) was defined as the time from diagnosis to death by any cause. The survival analysis for EFS and OS was done using the Kaplan–Meier method. Those lost to follow-up were censored for the survival analysis at the time of the last follow-up. We also compared the clinical data regarding the diagnostic delays, tumour size at presentation and the histology between the two groups: those undergoing orchiopexy and those not.

## Results

Out of a database of 740 patients with testicular tumours treated from 2014 to 2020, 54 patients were identified with undescended testis and testicular tumours. The mean age was 32.4 years (median age 32, range: 15–56 years). Seventeen patients (31.4%) had prior orchidopexy, and 37 (68.6%) presented with testicular cancer in uncorrected cryptorchid testis (Table 1). The median age at orchidopexy was 13.5 years (range: 2–32 years). At the time of diagnosis, the contralateral testis was abnormal in 15 patients (27.7%), viz. being undescended in 11 patients, sonographically abnormal with microlithiasis in 3 patients and atrophic in 1 patient. None developed testicular cancer as a part of a clinical syndrome or had a family history of testicular cancer. One patient was HIV sero-positive at the time of diagnosis.

The median time from the onset of symptoms to diagnosis was 2 months (range: 1–36 months). The time from presentation to our hospital to the beginning of treatment was delayed by more than 1 month in 13 patients. The longest delay was 4 months. The delays were due to the establishment of diagnosis in patients who had not undergone orchiopexy. Two of these patients were initially misdiagnosed to have gastrointestinal malignancies: one as pancreatic cancer and the other as gastrointestinal stromal tumour (GIST). 33 (61.1%) patients had seminoma and 21 (38.9%) patients had non-seminomatous germ cell tumours. Seminomas were more common in those with uncorrected undescended testes (75.67%) versus those who had undergone orchiopexy (23.52%). Overall, 19 patients had metastatic disease at presentation – lung metastasis was present in 7 patients, non-regional lymph node metastasis in 5 patients and non-pulmonary visceral metastases in 7 patients. Thirty (55.5%) patients underwent orchidectomy upfront while in 22 (40.7%) patients, orchidectomy was done after chemotherapy. The surgical approach included high inguinal orchidectomy, exploratory laparotomy or laparoscopic surgery per the clinical situation. Orchidectomy was not done in two patients due to disseminated disease, including brain metastasis. One had complete metabolic response (CMR) after chemotherapy (seminoma) and was kept on observation.

Nine patients with prior orchiopexy underwent high inguinal orchidectomy upfront. 20 (34.14%) patients had testis located in the pelvis, 8 (9.7%) patients had testis located in the upper abdomen and 9 patients had inguinal masses: they required laparotomy/ laparoscopy for orchidectomy. Thirteen patients had masses with vascular or ureteric encasement, requiring complex surgeries with vascular cover.

Fifty of the 54 patients received chemotherapy, and only four could be spared chemotherapy due to stage 1 disease, one of whom received para-aortic radiation. The other three were kept on active surveillance after orchidectomy.

Sixteen patients underwent RPLND for significant residual after chemotherapy. Post RPLND, nine patients did not have viable residual disease (necrosis/fibrosis), two had residual disease, three had mature teratoma and one had a teratoma with a somatic malignancy (adenocarcinoma).

**Figure 2.**
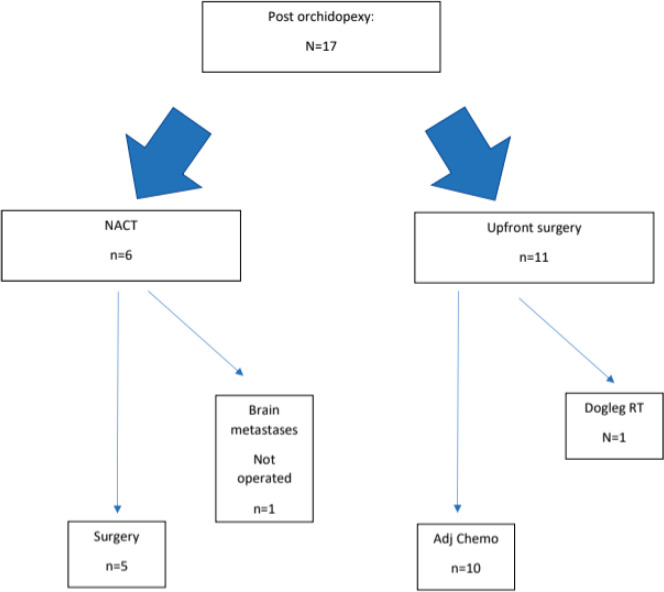
Treatment flowchart for patients who had undergone prior orchidopexy.

**Figure 3.**
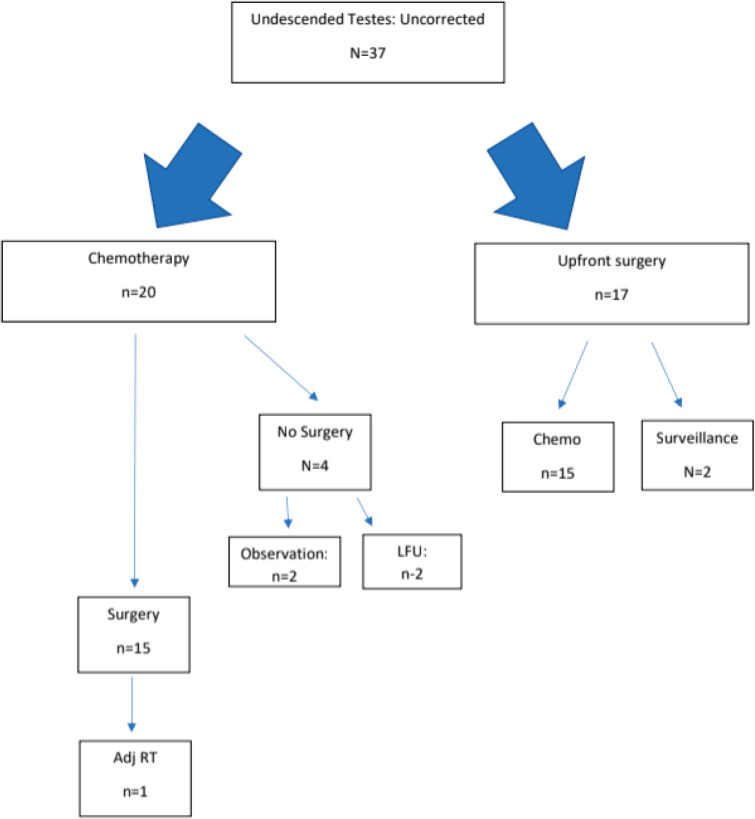
Treatment flowchart for patients who had not undergone prior orchidopexy.

Figure 2 depicts the sequence of therapies received by patients who had undergone prior orchidopexy. Figure 3 depicts the sequence of therapies received by patients who had not undergone prior orchidopexy. The chemotherapy regimen and the RPLND rates are summarised in Table 2.

The median follow-up for these patients was 66 months (95% CI: 51–76). There were four patients (all NSGCT) who relapsed after front-line therapy. The recurrences occurred at 1, 4, 4 and 19 months from the last treatment. Three patients received salvage chemotherapy while one was given only best supportive care alone at relapse, given his poor performance status. Of the three patients treated with salvage chemotherapy, one patient was treated with Gemcitabine-Oxaliplatin, and two were treated with a VIP regimen. Two were salvaged with second-line chemotherapy, and one was asymptomatic with residual disease at the last follow-up. One patient (NSGCT) had unresectable disease post-first line chemotherapy with rising tumour markers. Salvage chemotherapy with Gemcitabine-Paclitaxel was started. However, he succumbed to the disease.

**Figure 4.**
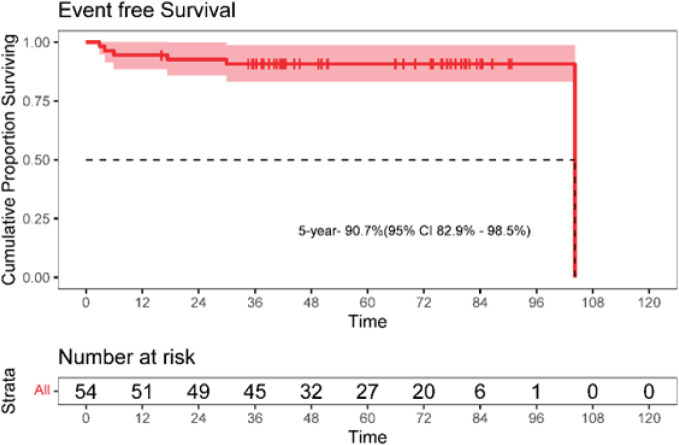
Kaplan-Meier curve for event-free survival (EFS).

**Figure 5.**
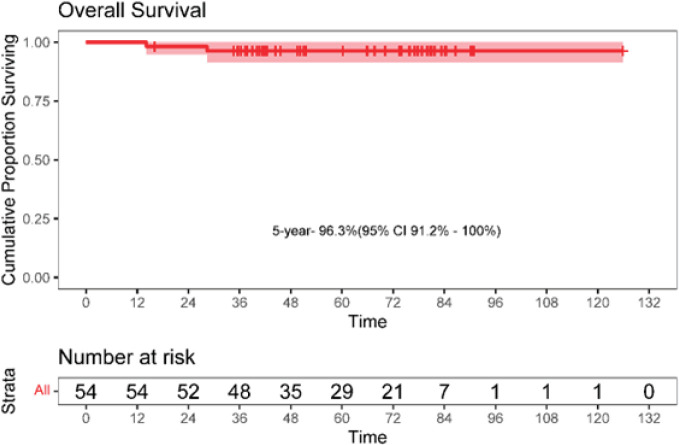
Kaplan-Meier curve for overall survival (OS).

The 5-year EFS was 90.7% (95% CI: 82.9–98.7). The 5-year OS was 96.3% (95% CI: 91.2–100). The Kaplan–Meier survival curves for EFS and OS are depicted in Figures 4 and 5.

## Discussion

Cryptorchidism inherently predisposes to the development of cancer as a part of the broader syndrome of testicular dysgenesis. The risk of development of testicular cancers subsequently in the cryptorchid testis can be reduced to some extent by orchiopexy. Our study highlights the challenges associated with the management of undescended testis and also the use of neoadjuvant chemotherapy with the view of avoiding aggressive morbid surgeries. By dividing the testicular cancers occurring in undescended testis into patients undergoing orchiopexy or not undergoing orchiopexy, we have tried to highlight the impact of orchiopexy on the outcomes of testicular tumours if and when they occur in an orchiopexy testis. We found that prior orchiopexy impacts not only early detection of the testis but also the tumour’s bulkiness, stage of presentation, difficulty in the diagnosis of cancer and, therefore, even delays in treatment. Those who had not undergone orchidopexy had a larger tumour size at presentation (*p* = 0.0007). Undescended testis constituted 7.3% of all testicular cancers, matching the incidence of 5%–10% reported by Petersson *et al* [12]. Thyavihally *et al* [11] reported similar results in an abstract and showed that testicular tumours in undescended testis presented in an advanced stage. They also suggested that upfront surgery for undescended testis is morbid and requires neoadjuvant chemotherapy. Surgical removal of the primary tumour in an undescended testis with bulky metastasis is difficult. These tumours usually require initial chemotherapy followed by surgical removal of the primary and the residual metastasis. Atypically altered ilioinguinal metastases may necessitate changing radiotherapy ports RPLND boundaries [9]. The advanced-stage at presentation of testicular cancers in undescended testis has also been demonstrated by Saini *et al* [10]. The advanced stage at presentation has been uniformly described as a poor prognostic factor with 5-year survival ranging from 48% to 65% [8, 13, 14]. Advanced stages also mean increased requirement of intense chemotherapy four # BEP or VIP chemotherapy, with its accompanying long-term side effects.

Patients with intra-abdominally located testis may require a laparotomy or a laparoscopic surgery compared to the relatively simple procedure of high inguinal orchidectomy.

Correction of undescended testis makes the diagnosis of cancer easy and avoids unnecessary delays in diagnosis and prompts initiation of therapy. In patients who had not undergone orchidopexy, the time from presentation to our hospital to the beginning of treatment was delayed by more than 1 month in 13 out of 36 (36.1%) The most extended delay was 4 months. Delays in diagnosing and treating testicular cancer have been associated with inferior long-term survival [15]. Among those who had undergone prior orchidopexy, no patient had a delay in diagnosis of more than 1 month.

Intra-abdominally located and intra-pelvic testes also cause significant diagnostic difficulties. In our series, two patients with intra-abdominally located testis were initially registered under the gastrointestinal disease management group (DMG). One had an fine-needle aspiration cytology (FNAC)-based diagnosis of ‘*adenocarcinoma of the pancreas’,* and the other had a diagnosis of GIST based on undifferentiated morphology and c-kit (CD 117) positivity [16] and was later diagnosed as Seminoma. Patients with cryptorchidism present at a relatively later age (up to 56 years) when germ cell tumours may not be considered in differentials. This may further confound and contribute to delays in diagnosis.

Other results of this study are similar to those reported in other publications on the subject. The median age at presentation was 32 years, the usual age for the presentation of testicular tumours. Seminomas were commoner in the uncorrected group, as seen in other studies [8]. Nine patients had abnormal contra-lateral testis and stood to have a higher risk of cancer compared to the general population [17].

The earliest time of orchiopexy in this series, after which testicular cancer developed, was 2 years. The ideal time of orchiopexy is 6–12 months, and current recommendations suggest that the orchiopexy must be done before 18 months [18]. In a Swedish population-based study, the mean age at orchiopexy was 8.6 ± 3.5 years [10]. Due to several reasons, the practical age at which orchiopexy is done is 4–5 years. Though this procedure may not help in the prevention of testicular cancer, we have shown here that orchiopexy also has a favourable role in screening and early detection, as well as influencing a good outcome on testicular cancers.

Testicular cancers occurring in uncorrected cryptorchid testes were associated with significantly higher rates of vascular and ureteric encasement, which might become a significant problem if related to significant post-chemotherapy residual and bulky disease. Complex vascular reconstructive procedures [19] are associated with higher morbidity and recurrence rates and require referral to specialised centres. Upfront chemotherapy may be preferred before such complex surgeries and simplify the treatment [20]. Upfront chemotherapy in these situations also leads to the quick institution of treatment of abdominal and pulmonary symptoms, which may quickly become life-threatening [20–22].

The observations made in our study are limited by the retrospective nature of the data and the few events occurring in the cohort. However, to the best of authors’ knowledge, we note that this is the largest cohort of patients with testicular cancer developing in the undescended testis in the literature from India. Our cohort’s OS and outcomes matched that of patients with normally descended testes seen in other studies.

## Conclusion

From our experience of managing germ cell tumours occurring in undescended testis, we have shown that therapy outcomes in these patients approach those of patients with customarily descended testes. However, they often present late for therapy, with larger primary tumours, particularly those without prior orchiopexy. These patients require complex multimodality management. The effort is rewarding, with gratifying results. In the first such series from India, we show that testicular tumours in the cryptorchid are also as curable as the germ cell tumours developing in the descended testis. However, ours is a high-volume tertiary care centre with ease of access to multimodality facilities including surgical oncology expertise and critical care. A

multidisciplinary DMG with expertise in managing complex cases is crucial for a favourable outcome in these groups of patients. This may be one of the reasons for our better outcomes. We have also shown that the tumours developing in an orchiopexed testis tended to be smaller in size. Thus, orchiopexy may confer a favourable outcome and simplifies further management, should a patient with undescended testis develop testicular cancer.

## Conflicts of interest

None.

## Financial declaration

None of the authors have any funding sources to declare pertaining to present work.

## Figures and Tables

**Table 1.** Baseline characteristics.

		Total (n = 54) Number(%)
Seminoma	Non Seminoma	21 (38.9)
	Seminoma	33 (61.1)
T stage	Tx	35 (64.8)
	T1	10 (19.6)
	T2	7 (13.7)
	T3/T4	2 (3.9)
N stage	N0	13 (24.1)
	N+	41 (75.9)
M Stage	M0	35 (64.8)
	M1a	12 (22.2)
	M1b	7 (13.0)
Tumour marker risk	S0	21 (38.9)
	S1	15 (27.8)
	S2	11 (20.4)
	S3	7 (13.0)
IGCCC RISK	GOOD	32 (66.7)
		9 (18.8)
	POOR	7 (14.6)
	NA	6(11.1)
Time from presentation to start of Rx in months	<1	41 (75.9)
	>=1	13 (24.1)
Size of the primary tumour(longest diameter, cm)	median [iqr]	8.2 [ 7.1, 10.5]

**Table 2:** Showing treatment received by Seminoma and NSGCT

Seminoma (n = 33) Treatment received: Number (%)	NSGCT (n = 21) Treatment received: Number(%)
3# BEP: 14 (42.2) 4# of EP: 5 (15.1) Carboplatin (AUC-7): 8 (24.2) Para-aortic RT: 1 (3.0) 2# BEP+ 2 EP: 1 (3.0) 4#VIP: 2 (6.0)	4# EP: 1 (4.76) 4# of BEP: 13 (61.9) 4#VIP: 3 (14.28) 4#TIP: 1(4.76) 2# BEP: 1(4.76) 3# BEP: 1 (4.76)
4 patients underwent RPLND (12.12%)	12 patients required RPLND (57.14%)


AUC = Area under the curve; RPLND: Retroperitoneal lymph node dissection.
